# Associations between fat distribution and obstructive sleep apnea severity among individuals with type 2 diabetes: an MRI-based study

**DOI:** 10.1038/s41598-026-58058-0

**Published:** 2026-06-22

**Authors:** Kim Ahtola, Martin Ulander, Jonas Agholme, Farida Amiri, Pontus Henriksson, Olof Dahlqvist Leinhard, Peter Lundberg, Nils Dahlström, Carl-Johan Carlhäll, Stergios Kechagias, Patrik Nasr, Mikael Forsgren, Mattias Ekstedt, Fredrik Iredahl

**Affiliations:** 1 Department of Health, Medicine and Caring Sciences, Linköping University, Linköping, Sweden; 2 Department of Biomedical and Clinical Sciences, Division of Neurobiology, Faculty of Medicine, Linköping University, Linköping, Sweden; 3 Department of Clinical Neurophysiology, Linköping University Hospital, Linköping, Sweden; 4 Primary Health Care Center Åby, Department of Health, Medicine and Caring Sciences, Linköping University, Linköping, Sweden; 5 Center for Medical Image Science and Visualization (CMIV), Linköping University, Linköping, Sweden; 6 AMRA Medical AB, Linköping, Sweden; 7 Wallenberg Center for Molecular Medicine (WCMM), Linköping University, Linköping, Sweden; 8 Department of Gastroenterology and Hepatology, Department of Health, Medicine and Caring Sciences, Linköping University, Linköping, Sweden; 9 Department of Radiation Physics, Department of Health, Medicine and Caring Sciences, Linköping University, Linköping, Sweden; 10 Department of Clinical Physiology in Linköping, Department of Health, Medicine and Caring Sciences, Linköping University, Linköping, Sweden; 11 Department of Radiology, Department of Health, Medicine and Caring Sciences, Linköping University, Linköping, Sweden

**Keywords:** Sleep-disordered breathing, Central obesity, Abdominal adiposity, Insulin resistance, Polygraphy, Sex-specific risk factors., Diseases, Endocrinology, Health care, Medical research, Risk factors

## Abstract

Obstructive Sleep Apnea (OSA) affects 1 billion people globally, with a male-to-female ratio of 2:1. While obesity is a major risk factor, sex differences in fat distribution may influence OSA risk. This study examined the relationship between visceral (VAT), subcutaneous (ASAT), and total abdominal fat (TAAT) and OSA severity in patients with type 2 diabetes (T2DM). We enrolled 164 T2DM patients (97 males, 67 females) from the EPSONIP-Sleep study; 151 had complete MRI and polygraphy data. OSA severity was evaluated by home respiratory polygraphy. Linear regression assessed AHI, logistic regression assessed moderate-to-severe OSA, and the Kruskal–Wallis test compared AHI across VAT-z/ASAT-z strata. In males, VAT, ASAT, and TAAT were associated with AHI in unadjusted analyses (β = 1.3, *p* = 0.009; β = 1.5, *p* = 0.010; β = 0.85, *p* = 0.009, respectively). ASAT (OR = 1.23/L, 95% CI 1.01–1.49, *p* = 0.049) and TAAT (OR = 1.10/L, 95% CI 1.00–1.22, *p* = 0.02), but not VAT, were associated with moderate-to-severe OSA. No statistically significant associations were detected in females. Associations were attenuated after BMI adjustment. AHI did not differ across VAT-z/ASAT-z strata (*p* = 0.430). Sex differences influenced the relationship between fat and OSA severity. In males, abdominal fat volumes were associated with higher OSA severity in unadjusted analyses, but not after BMI adjustment. No statistically significant associations were detected in females. These findings should be interpreted cautiously given the modest sample size.

**Supplementary Information:**

The online version contains supplementary material available at 10.1038/s41598-026-58058-0.

## Introduction

 Obstructive Sleep Apnea (OSA) is a chronic disorder characterized by repeated upper airway obstruction during sleep, resulting in impaired ventilation and oxygen desaturation. The prevalence of OSA varies depending on diagnostic criteria, with estimates ranging from 9% to 38%^1^, affecting nearly 1 billion people globally^2^. Although obesity is a significant risk factor, not all obese individuals develop OSA; conversely, OSA can occur in non-obese populations^3–5^. OSA is approximately twice as prevalent in men compared to women, possibly due to hormonal factors, anatomical differences, and distinct fat distribution patterns – men typically accumulate more visceral fat, while women predominantly store subcutaneous fat^6^.

Fat distribution plays a critical role in the pathophysiology of OSA. Fat accumulation in the neck and upper airway contributes to pharyngeal narrowing, increasing the risk of airway collapse during sleep. Additionally, abdominal obesity may increase mechanical loading, reduce lung volume, and promote upper-airway collapsibility during sleep^7,8^. Visceral adiposity is strongly associated with cardiometabolic risks, such as atherosclerosis and Metabolic dysfunction-Associated Steatotic Liver Disease (MASLD), whereas gluteofemoral fat may confer protective metabolic effects^3,4,9–12^.

Techniques such as dual-energy X-ray absorptiometry (DXA), bioelectrical impedance analysis (BIA), and computed tomography (CT) have commonly been employed to assess fat distribution in clinical and epidemiological research^13–15^.

The link between visceral adiposity and OSA is well-documented, with associations generally more pronounced in men than women^13^. Furthermore, the link between OSA and metabolic liver disease, particularly MASLD, is well-documented^3^. However, studies specifically examining detailed fat distribution in OSA populations, especially among individuals with Type 2 Diabetes Mellitus (T2DM), remain limited.

Magnetic resonance imaging (MRI) is the gold standard for analyzing fat distribution^14,16^, but its use in OSA research has been scarce. MRI-based assessment allows precise quantification of abdominal fat depots and can be used to examine whether depot-specific fat distribution is associated with OSA severity, particularly given known sex differences in fat distribution and OSA prevalence^17,18^.

Although previous studies have reported associations between OSA and visceral adiposity^13,18^, no studies have specifically utilized MRI to examine detailed fat distribution in relation to OSA severity among patients with T2DM.

This study aimed to investigate the relationship between MRI-quantified abdominal fat compartments, particularly visceral adipose tissue (VAT) and abdominal subcutaneous adipose tissue (ASAT), and OSA severity in patients with T2DM, and to assess whether any observed associations persisted after accounting for general adiposity.

## Materials and methods

### Study protocol

This project is based on EPSONIP-Sleep, a sub-study within EPSONIP (Evaluating the Prevalence and Severity of NAFLD in Primary Care)^19^. EPSONIP is a prospective cohort investigating Metabolic dysfunction-Associated Steatotic Liver Disease (MASLD), formerly known as Non-Alcoholic Fatty Liver Disease (NAFLD), in patients with T2DM using MRI in Swedish primary care^19^. Research participants were randomly recruited from five primary healthcare centers in Southeast Sweden during their annual check-up appointments, with enrollment taking place between March 2019 and October 2023. Inclusion criteria were a diagnosis of T2DM and age between 35 and 75 years old. Exclusion criteria were inability to undergo MRI (claustrophobia, pacemaker, metal implants, extreme obesity and/or pregnancy), alcohol dependence, previously diagnosed liver cirrhosis or chronic liver disease (except MASLD). The EPSONIP study, including the sub study EPSONIP-Sleep, was conducted in accordance with the Declaration of Helsinki and approved by the Regional Ethical Board of Östergötland (EPSONIP: 2018/176 − 31 and 2018/494 − 32, EPSONIP-Sleep: 2019–03854). All participants provided written informed consent prior to inclusion. The study was registered at clinicaltrials.gov (identifier NCT03864510). Linköping University is research principal (2019–03854).

All research participants in the EPSONIP study were invited to participate in EPSONIP-Sleep, with the only exclusion criteria being the presence of an implantable cardioverter defibrillator.

Research participants underwent home respiratory polygraphy for assessment of OSA (Nox T3, Nox Medical, Iceland). The Nox T3 has previously shown good agreement with polysomnography for detecting OSA^20^. Research participants were trained by research staff on how to set up the device. The device was used to monitor nasal airflow, heart rate, blood oxygen levels, thoracic and abdominal respiratory movements (by respiratory inductance plethysmography), body position, and snoring sound (via sound recording)^21^. Apnea was defined as a ≥ 90% reduction in airflow for at least 10 s, while hypopnea was a ≥ 30% reduction in airflow for at least 10 s, with ≥ 3% blood oxygen desaturation.

The Apnea-Hypopnea Index (AHI; events/h) was the number of apneas + hypopneas divided by estimated sleep time. The oxygen desaturation index (ODI; events/h) was the number of ≥ 3% desaturations per hour of estimated sleep time. Because home respiratory polygraphy does not directly measure sleep, estimated sleep time based on actigraphy and sleep logs was used in accordance with the study protocol. This approach was chosen to reduce overestimation of sleep time compared with total recording time, although some underestimation or misclassification of sleep time remains possible.

Research participants with an AHI ≥ 5 events/h were classified as having OSA and were further categorized by severity as mild (AHI 5–15 events/h), moderate (15–30 events/h), or severe (≥ 30 events/h)^21^.

Average nocturnal oxygen saturation was computed as the area under the saturation-time curve (trapezoidal rule) divided by total sleep time.

### Measurement of body fat distribution

Research participants underwent neck-to-knee imaging using Achieve dStream 1.5 T MR scanners (Philips Healthcare, The Netherlands), providing volumetric data on VAT, ASAT, as well as liver fat fraction (proton density fat fraction; PDFF), and muscle composition. The image data were processed and quantified using AMRA^®^ researcher (AMRA Medical AB, Linköping, Sweden). AMRA developed the MRI protocol and performed body composition analysis in the UK Biobank; the same methods were used in this study^16,22^.

MRI-derived z-scores were computed using matched virtual control groups (VCG**)**. These reference distributions were used as a standardized framework for relative body composition phenotyping; however, because they were derived from the UK Biobank rather than a Swedish T2DM cohort, their applicability should be interpreted with some caution. For each participant, ASAT-z and VAT-z were referenced to the distribution of VCG controls matched on sex and BMI within ± 1 kg/m²; if < 150 matches were available, the BMI window was symmetrically widened in 0.1 kg/m² steps until ≥ 150 controls were included. VAT and ASAT volumes (L) were normalized by height² (m²) to L/m² before z-score calculation^22^.

Inspired by previous studies, such as Tejani et al.^15^ and Linge et al.^22^ VAT-z and ASAT-z were dichotomized as high (≥ 0) or low (< 0). Participants were stratified into four groups: high/high, high/low, low/high, and low/low (VAT-z/ASAT-z) to examine how depot patterns relate to OSA severity.

In addition to z-scores, we analyzed volumetric total abdominal adipose tissue (TAAT = VAT + ASAT, in liters) as a global burden metric potentially reflecting mechanical load on respiratory function.

### Statistical analysis

All statistical analyses were performed using R version 4.3.3 (The R Foundation for Statistical Computing, Vienna, Austria). Descriptive statistics were used to summarize the data, reporting means and standard deviations (SD) for continuous variables. Welch’s t-test was employed to compare group means where variance equality could not be assumed. Linear regression was used to examine relationships between continuous variables, while logistic regression was applied to analyze associations with binary outcomes. AHI distributions across the four VAT-z/ASAT-z strata were compared using the Kruskal–Wallis test.

Statistical significance was set at *p* < 0.05. The Apnea-Hypopnea Index (AHI) was the primary outcome of interest in all regression analyses, as it is the most widely accepted and clinically relevant measure of OSA severity.

To support and contextualize the main findings, we also performed exploratory analyses using additional indices of nocturnal hypoxemia: average oxygen saturation during sleep (AvSat), the proportion of sleep spent with oxygen saturation below 90% (T90), and the Oxygen Desaturation Index (ODI). These supplementary outcomes were analyzed using the same regression framework and are presented in Supplementary Table 2.

We conducted an a priori power appraisal. Detecting a small effect (Cohen’s f²=0.02) in simple linear regression at α = 0.05 and 80% power would require approximately 395 participants. For medium effects (f²=0.15), approximately 54 participants are needed; for logistic models with medium effects (Cohen’s h = 0.5), approximately 63 per group.

We included 151 research participants in the primary complete-case AHI analyses (91 males and 60 females), providing adequate power for medium-sized effects. We acknowledge reduced power for small effects and within-sex strata (particularly in females). Primary models were run unadjusted and adjusted for BMI to examine whether associations between depot measures and OSA severity persisted after accounting for overall adiposity. Because the primary aim was to assess whether MRI-derived depot measures were associated with OSA severity beyond general adiposity, BMI was selected as the key adjustment variable. Given the modest sample size, the sex-stratified analyses, and the limited number of events in the logistic models, broader multivariable adjustment was not performed in the primary analyses in order to reduce the risk of overfitting and unstable estimates.

## Results

**Table 1:**
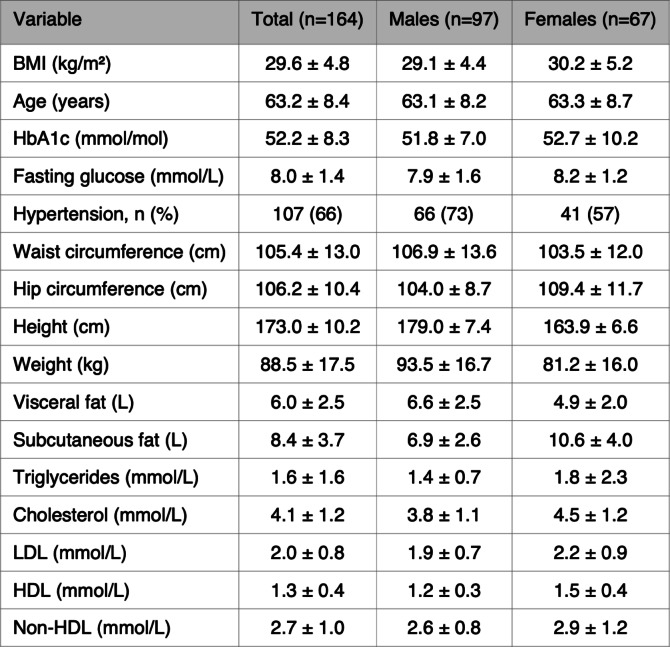
Demographics and clinical characteristics of study population. Fat volumes (VAT, ASAT) are expressed in liters (L) All anthropometric measures are expressed in cm or kg as indicated.

**Fig. 1:**
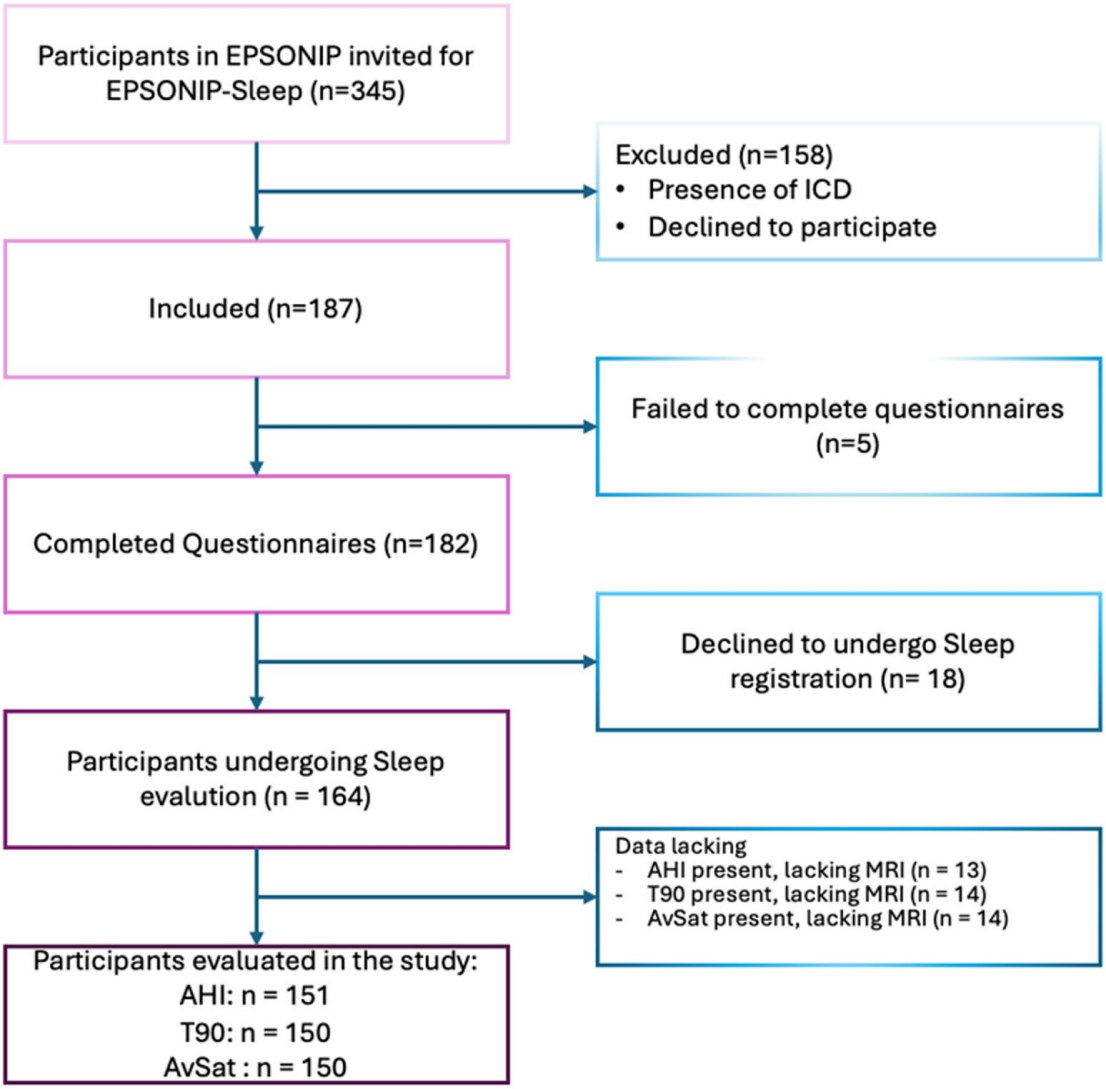
Flow diagram of EPSONIP-Sleep inclusion. Participant flow: 345 invited → 164 completed MRI + respiratory polygraphy → 151 included in primary AHI analyses. Complete-case analyses were used. Sample sizes for ODI, average saturation, and T90 are reported in Supplementary Table 2. Abbreviations: AvSat, average saturation; T90, time with saturation < 90%; AHI, apnea–hypopnea index.

As illustrated in Fig. 1 and 345 individuals were invited and 164 underwent both MRI and overnight polygraphy. Of these, 13 lacked usable MRI results, leaving 151 participants with complete MRI and AHI data for the primary analyses. Complete-case analyses were used. Sample sizes for secondary outcomes (ODI, average saturation, and T90) differed because of missing data and are reported in Supplementary Table 2. Reasons for exclusion at each step are detailed in Figure 1.

The study population consisted of 164 research participants (97 males and 67 females) with a mean age of 63.2 ± 8.4 years (Table 1). The overall BMI was 29.6 ± 4.8 kg/m². Males had larger waist circumferences (106.9 ± 13.6 cm) compared to females (103.5 ± 12.0 cm), while females exhibited larger hip circumferences (109.4 ± 11.7 cm) than males (104.0 ± 8.7 cm). Antihyperglycemic and cardiometabolic medications are summarized in Supplementary Table 3. Hypertension was present in 107 participants, with 66 males and 41 females diagnosed. Descriptive statistics for BMI, VAT-z, ASAT-z, and TAAT across AHI categories are provided in Supplementary Table 1.

**Fig. 2:**
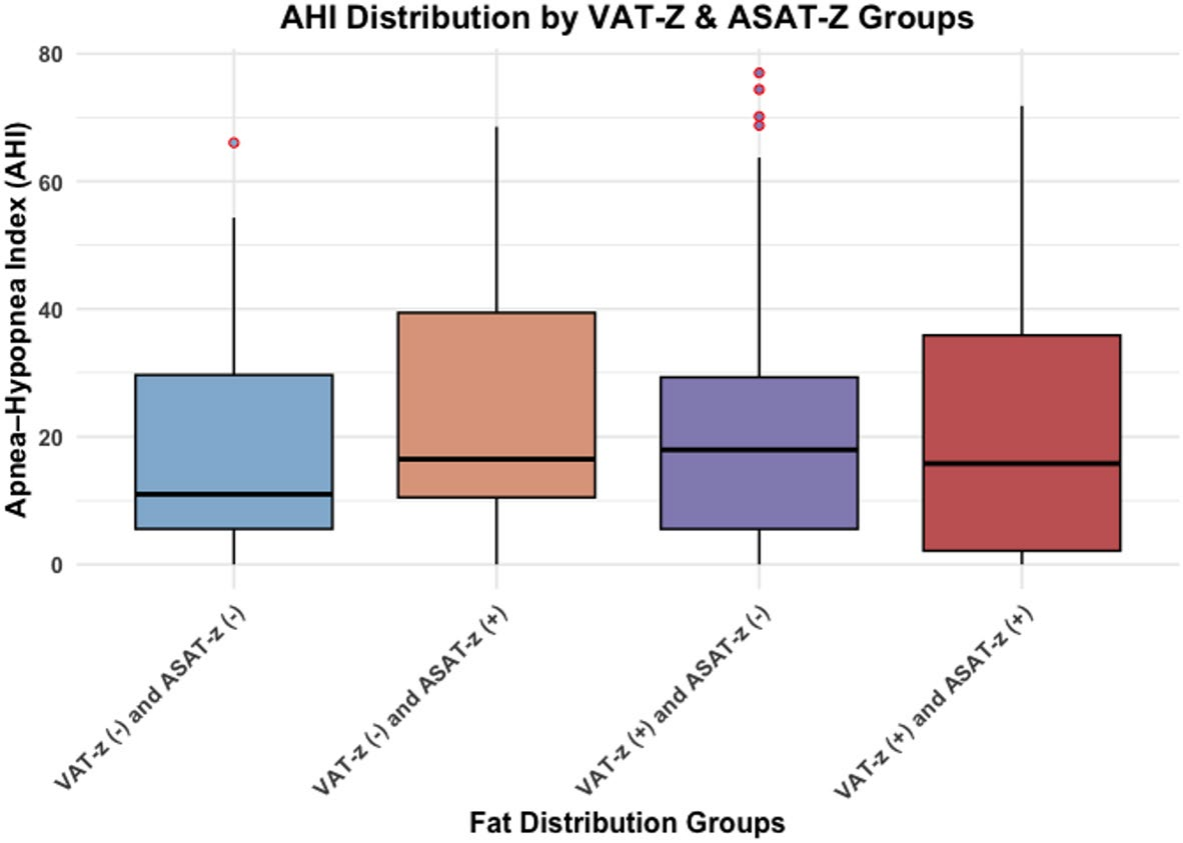
AHI across VAT-z/ASAT-z strata (high ≥ 0 vs. low < 0). Group sizes were high/high, *n* = 46; high/low, *n* = 59; low/high, *n* = 23; and low/low, *n* = 23. Boxes show the median and IQR; whiskers denote 1.5×IQR. Groups did not differ (Kruskal–Wallis χ²=2.76, df = 3, *p* = 0.430). (+) z-score ≥ 0; (−) z-score < 0.

Overall, 67.5% met criteria for OSA (AHI ≥ 5). Moderate-to-severe OSA (AHI ≥ 15) was present in 31.1% of the 164 participants with AHI data. In the complete-case sample, moderate-to-severe OSA was more prevalent in males than females (37.4% vs. 18.3%; χ² *p* = 0.020). VAT-z and ASAT-z were not associated with AHI (linear regression: VAT-z β = 0.75, *p* = 0.396; ASAT-z β=−0.11, *p* = 0.913). The four VAT-z/ASAT-z strata included 46 participants in the high/high group, 59 in the high/low group, 23 in the low/high group, and 23 in the low/low group. AHI distributions did not differ across the strata (Kruskal–Wallis χ²=2.76, df = 3, *p* = 0.430; Fig. 2).

Unadjusted volumetric analyses showed significant associations with AHI in males, whereas no statistically significant associations were detected in females. In males: ASAT β = 1.5 events/h per liter (*p* = 0.010), VAT β = 1.3 events/h per liter (*p* = 0.009), TAAT β = 0.85 events/h per liter (*p* = 0.009). In females: ASAT β=−0.38 events/h per liter (*p* = 0.325), VAT β = 0.80 events/h per liter (*p* = 0.325), TAAT β=−0.10 events/h per liter (*p* = 0.745).

Supplementary Figs. 1 and 2 illustrate the sex disparity in OSA severity, highlighting higher AHI distribution in males.

Interaction analyses further supported these findings, revealing that the relationships between VAT, ASAT, and TAAT and AHI were significantly modified by sex. Specifically, the association between ASAT and AHI differed by sex (*p* = 0.007), as did the association for TAAT (*p* = 0.03), indicating stronger effects in males. No significant sex interaction was observed for VAT (*p* = 0.65).

**Fig. 3:**
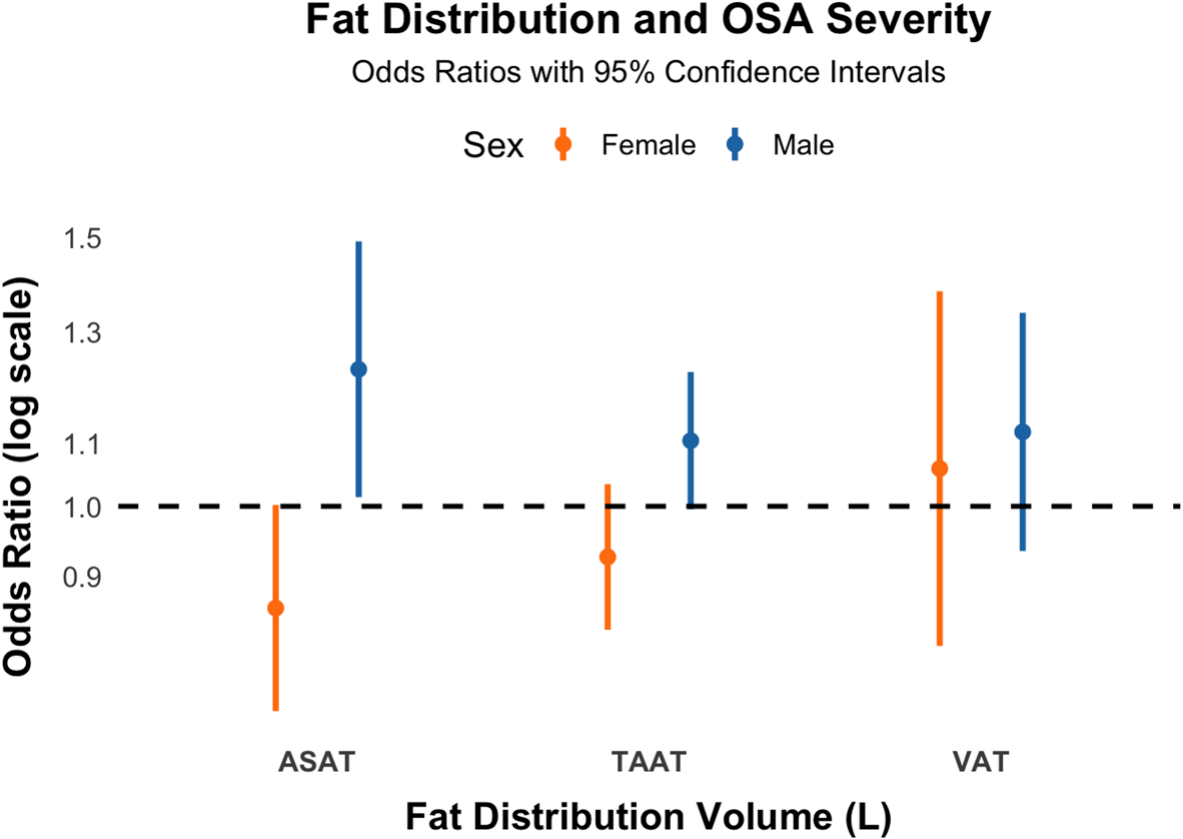
Odds ratios (95% CI) for moderate-to-severe OSA (AHI ≥ 15) per liter increase in ASAT, TAAT, and VAT, stratified by sex. In males, ASAT (OR 1.23, 95% CI 1.01–1.49) and TAAT (OR 1.10, 95% CI 1.00–1.22) were significant; VAT was not (OR 1.12, 95% CI 0.93–1.34). In females, no associations were significant (all 95% CI cross 1.0).

Logistic regression analyses partly supported these findings, identifying ASAT and TAAT, but not VAT, as significant predictors of moderate-to-severe OSA in males (Fig. 3). ASAT showed an odds ratio of 1.23 per liter (95% CI: 1.01–1.49, *p* = 0.049), VAT an odds ratio of 1.12 per liter (95% CI: 0.93–1.34, *p* = 0.22), and TAAT an odds ratio of 1.10 per liter (95% CI: 1.00-1.22, *p* = 0.02). In females, no statistically significant associations were detected for any of these measures (*p* > 0.8), as indicated by confidence intervals that included 1.0 in Fig. 3.

Logistic regression analyses were performed using moderate-to-severe OSA (AHI ≥ 15) as the outcome, since this threshold is more likely to reflect clinically relevant disease requiring intervention. When repeating the analyses using any OSA (AHI > 5) as outcome, the associations between fat distribution and OSA were weaker and not statistically significant. This supports the use of moderate-to-severe OSA as the more meaningful outcome in this context.

**Fig. 4:**
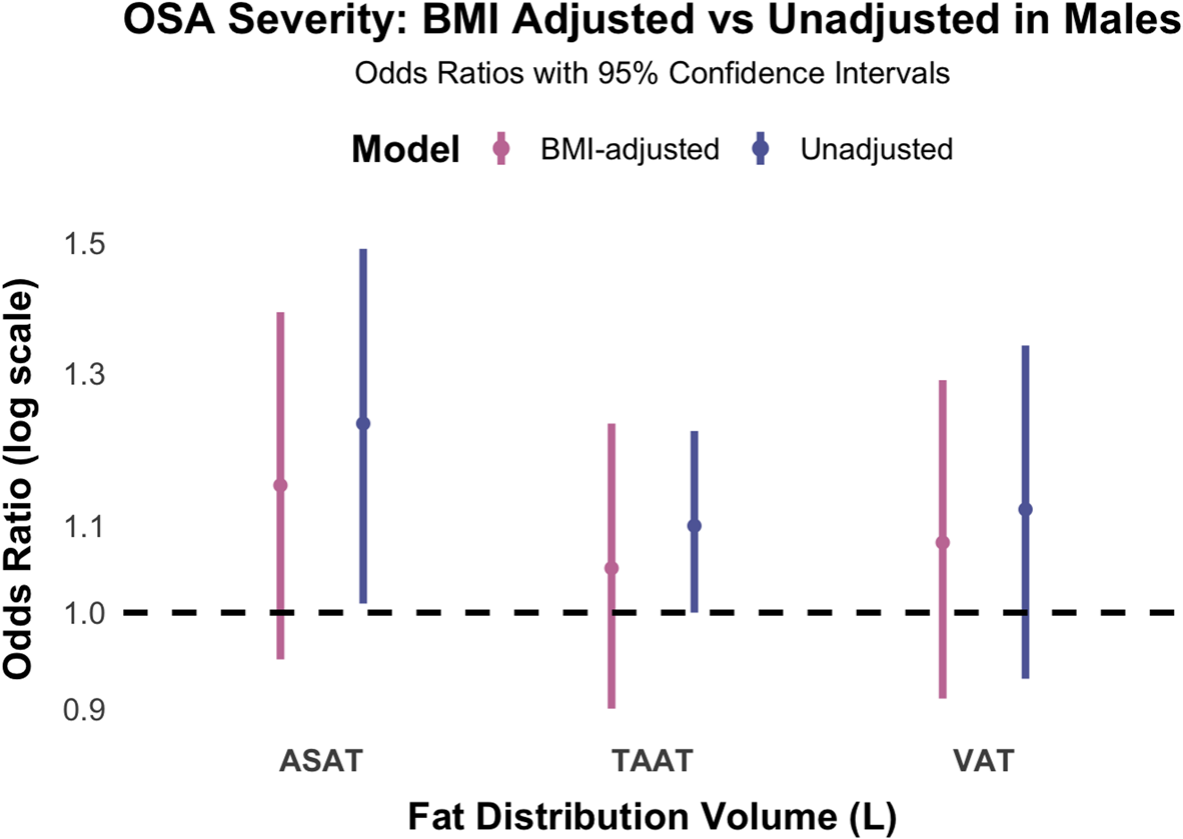
Unadjusted and BMI-adjusted odds ratios (95% CI) for AHI ≥ 15 per liter of ASAT, TAAT, and VAT in males. Associations significant in unadjusted models attenuated to non-significance after BMI adjustment.

In a subsequent analysis (Fig. 4), BMI adjustment was added to the logistic regression models in males. After BMI adjustment, the associations with ASAT and TAAT were attenuated and no longer statistically significant. The figure demonstrates that in the unadjusted models, ASAT and TAAT lie above the reference line (OR = 1.0) with CIs not crossing 1.0, whereas in the BMI-adjusted models, both fat volumes shift closer to the null and become non-significant. As exploratory analyses, we examined the associations between VAT, ASAT, and TAAT with T90 and average oxygen saturation. Several associations were attenuated after BMI adjustment; however, VAT remained associated with T90 and average saturation in males and with average saturation in females (Supplementary Table 2).

## Discussion

**Fig. 5:**
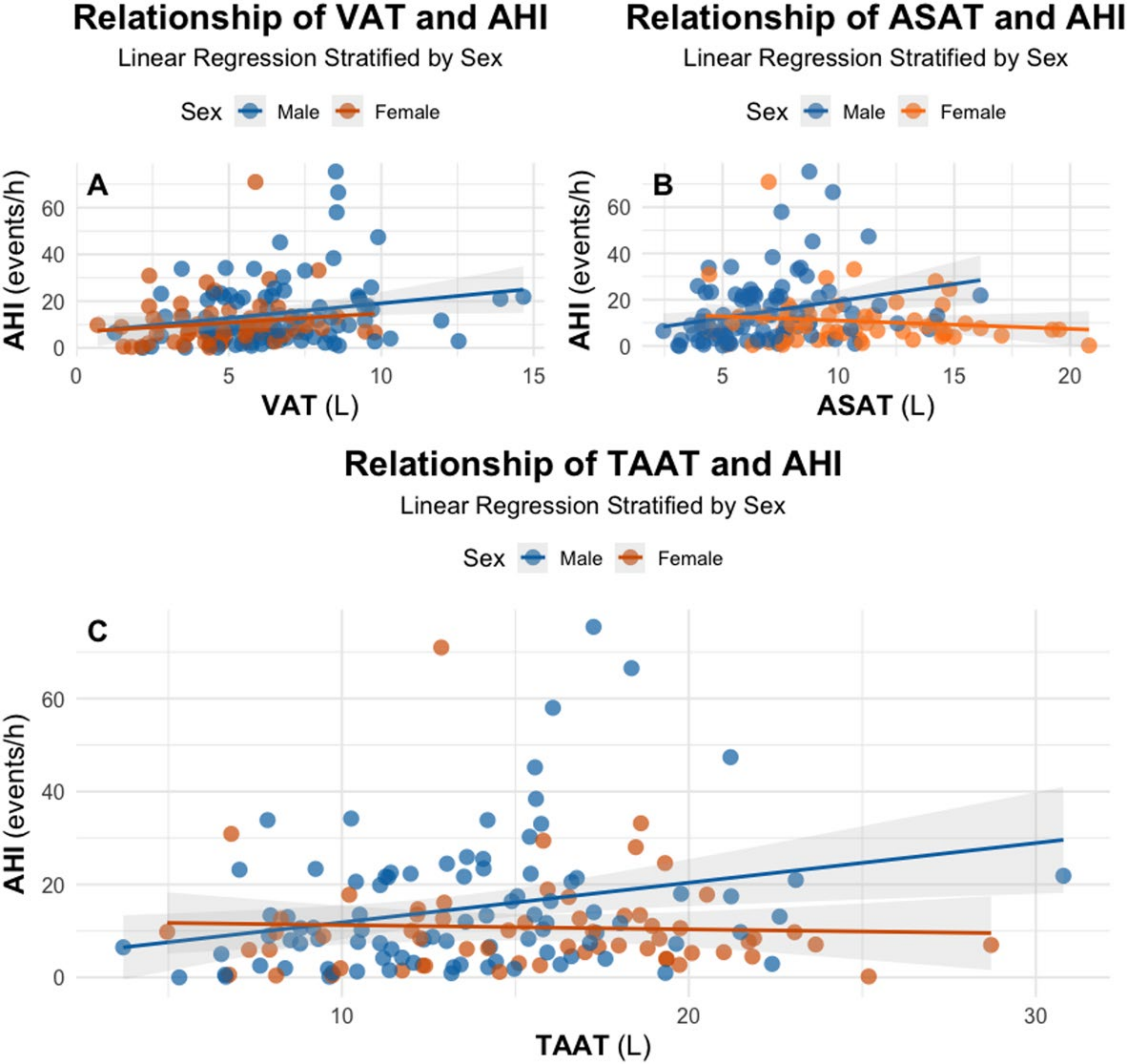
Linear associations of VAT (3 A), ASAT (3B), and TAAT (3 C) with AHI, stratified by sex. In males, higher VAT, ASAT, and TAAT were each associated with higher AHI (e.g., TAAT β = 0.85 events/h per liter, *p* = 0.009). In females, associations were not significant (e.g., TAAT β=−0.10 events/h per liter, *p* = 0.745).

In this cohort of patients with type 2 diabetes, abdominal fat volumes showed significant associations with OSA severity in males in unadjusted analyses (Fig. 5), but these associations did not remain statistically significant after adjustment for BMI. These findings did not demonstrate associations between compartment-specific abdominal fat measures and OSA severity independent of BMI in this cohort. MRI was nevertheless a methodological strength, as it enabled precise quantification of visceral and subcutaneous abdominal fat depots. To our knowledge, this is among the first studies to apply whole-body MRI to the study of fat distribution and OSA severity in individuals with type 2 diabetes. Regression analyses revealed significant associations between unadjusted fat volumes and AHI in males, particularly for ASAT and TAAT.

Logistic models were consistent: in males, ASAT and TAAT were associated with moderate-to-severe OSA (AHI ≥ 15), whereas no statistically significant associations were detected in females. BMI-adjusted logistic models attenuated these associations in males, rendering the relationships with ASAT and TAAT non-significant. Exploratory analyses of hypoxemia-related outcomes showed a partly different pattern, with persistent VAT associations for T90 and average saturation after BMI adjustment, particularly in males and for average saturation in females (Supplementary Table 2). These exploratory findings should be interpreted cautiously. MRI-derived volumes remain useful for detailed phenotyping and mechanistic investigation, even though they did not show clear independent associations beyond BMI in the present cohort.

Absolute fat volumes (ASAT, TAAT) were associated with OSA severity in males, whereas depot z-scores (VAT-z, ASAT-z) were not; this aligns with evidence that size-adjusted or standardized fat metrics can understate clinically relevant effects of absolute depot burden and distribution patterns^15,16,22,23^.

In the unadjusted analyses, ASAT and TAAT showed clearer associations with moderate-to-severe OSA in males than VAT, despite the a priori rationale for visceral adiposity as the more relevant depot. One possible explanation is that total abdominal fat burden, including subcutaneous fat, may better reflect the mechanical load on respiratory function and upper-airway stability than VAT alone. Experimental work has shown that abdominal compression can increase upper-airway collapsibility^7^ during sleep in obese men with OSA, supporting the idea that abdominal loading itself may be relevant. In addition, prior work has linked abdominal-region fat to OSA severity in men, suggesting that the relevant signal may not be limited to visceral fat alone^24^. More broadly, obesity-related OSA is thought to arise through several interacting mechanisms, including effects of adiposity on lung volume and pharyngeal collapsibility^8^. However, these interpretations should remain cautious, particularly because the observed associations attenuated after BMI adjustment. The limited number of moderate-to-severe cases also means that a VAT-specific association cannot be excluded.

This study underscores sex differences in the prevalence and predictors of OSA (AHI ≥ 15)^1,2,5,6,17,18^. In males, abdominal fat volumes were associated with OSA severity in unadjusted analyses, but these associations attenuated and were no longer statistically significant after BMI adjustment.

This pattern is broadly consistent with prior literature linking abdominal adiposity to OSA and cardiometabolic risk^9,10,12–14,18^. In females, no statistically significant associations were detected in the primary AHI-based analyses. Given the smaller female subgroup, these findings should be interpreted cautiously, as limited power may have reduced our ability to detect modest associations.

Unadjusted fat volumes, particularly ASAT and TAAT, showed the strongest associations in males, and interaction analyses suggested sex-related heterogeneity for ASAT and TAAT. No significant sex interaction was observed for VAT. The absence of statistically significant associations in females should be interpreted cautiously, as the smaller female subgroup limits power and precision. Larger studies are needed to determine whether this pattern reflects true sex differences or limited statistical power. Although MRI provided detailed and precise fat distribution measurements, its availability may be more limited in smaller hospitals and non-specialist settings, which may reduce its practicality for routine clinical use. Nevertheless, MRI allowed detailed compartment-specific fat assessment and helped examine whether abdominal fat depots were associated with OSA severity beyond general adiposity. This suggests a role for simpler, non-invasive alternatives in primary care. Anthropometric measures such as waist circumference may offer more accessible options for OSA risk stratification, especially in males with central obesity^14,15,17^.

Despite the strengths of this study, including MRI-based measurements and sex-stratified analyses, certain limitations must be acknowledged. Stratification by sex reduced the number of participants per group, which may have limited statistical power, particularly in females. The limited number of moderate-to-severe OSA cases, particularly among females, and potential collinearity between BMI and MRI-derived fat measures may also have reduced the precision of the estimates.

Residual confounding cannot be excluded, and factors such as age, diabetes-related characteristics, smoking, hypertension, and medication use may have influenced the observed associations. The use of complete-case analyses may also have introduced bias if participants with missing data differed systematically from those included in the primary analyses; this should be considered when interpreting the results. The cross-sectional design precludes causal inference, leaving open the question of whether fat accumulation leads to OSA or vice versa. Additionally, the neck-to-knee MRI scan did not include upper airway fat, which may also influence OSA severity. Finally, the use of cardiorespiratory polygraphy, while pragmatic, may underestimate OSA severity because it does not directly distinguish wake from sleep and may miss brief hypopneas. We attempted to mitigate this by estimating sleep time using actigraphy and sleep logs rather than total recording time^21^.

Future studies should build on these findings by incorporating longitudinal designs, more diverse populations, and larger female samples to clarify whether the absence of statistically significant associations in females reflects limited power, true sex differences, or both. If sex-specific differences are confirmed in larger studies, mechanistic factors could then be explored directly. Integrating sex-specific risk models and precision medicine approaches may help develop tailored screening and prevention strategies, especially for high-risk individuals in primary care settings. These efforts will deepen our understanding of the complex interplay between fat distribution and OSA and support more personalized care for patients with type 2 diabetes.

## Conclusion

This study found that associations of abdominal fat volumes with moderate-to-severe OSA in men were attenuated and no longer statistically significant after adjustment for BMI; independent associations beyond BMI were therefore not demonstrated in this cohort.

In females, no statistically significant associations were detected in the primary AHI-based analyses. Given the smaller female subgroup, this finding should be interpreted cautiously and may reflect limited statistical power, although true sex differences cannot be excluded. Exploratory analyses using alternative oxygenation measures (T90, AvSat) showed partly different patterns and should be interpreted cautiously.

## Supplementary Information

Below is the link to the electronic supplementary material.

Supplementary Material 1

## Data Availability

The datasets generated and analyzed during the current study are not publicly available due to the sensitive nature of personal data and the requirement for ethical approval from The Swedish Ethical Review Authority for the data to be legally shared but are available from the corresponding author on reasonable request and with a valid ethical permit.

## Abbreviations

T2DM: Type 2 Diabetes Mellitus
OSA: Obstructive Sleep Apnea
MRI: Magnetic Resonance Imaging
BMI: Body Mass Index
AHI: Apnea-Hypopnea Index
VAT: Visceral Adipose Tissue
ASAT: Abdominal Subcutaneous Adipose Tissue
TAAT: Total Abdominal Adipose Tissue
ASAT-z: Abdominal Subcutaneous Adipose Tissue z-Score
VAT-z: Visceral Adipose Tissue z-Score
MASLD: Metabolic dysfunction-Associated Steatotic Liver Disease
T90: Time under 90% saturation
ODI: Oxygen Desaturation Index
NAFLD: Non-Alcoholic Fatty Liver Disease
