# Supplementary material for: Associations between fat distribution and obstructive sleep apnea severity among individuals with type 2 diabetes: an MRI-based study
Source: Sci Rep. 2026 Jun 22;16:19329. doi: 10.1038/s41598-026-58058-0 (PMC13287766; doi:10.1038/s41598-026-58058-0)
Supplement: Supplementary file 1 — Supplementary Material 1 [file 41598_2026_58058_MOESM1_ESM.docx]

# Supplementary files


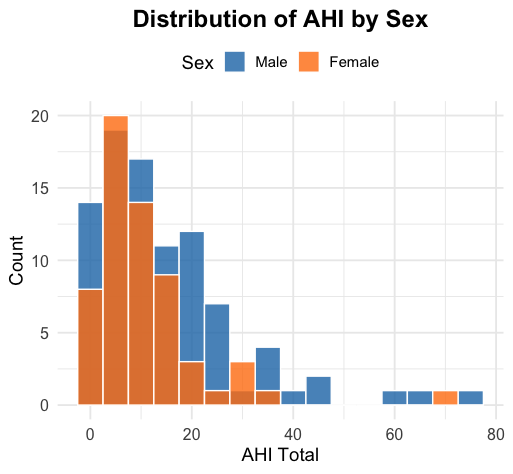


***Supplementary Figure 1.*** *Distribution of apnea-hypopnea index (AHI) by sex, showing greater OSA severity in males.*


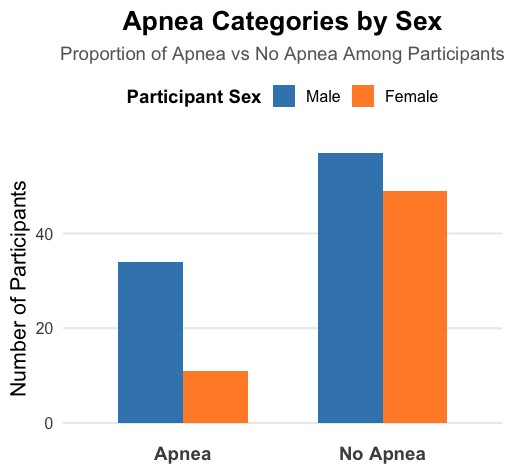


***Supplementary Figure 2.*** *Distribution of OSA and no-OSA categories by sex, showing a higher prevalence of OSA in males.*

# **Supplementary Table 1**: Supplementary Apnea category

| Summary Statistics by Variable and AHI Category | | | | | | | |
| --- | --- | --- | --- | --- | --- | --- | --- |
| Variable | AHI Category | Count | Mean | Standard Deviation | Median | Minimum | Maximum |
| BMI | No Apnea | 37 | 28.39 | 4.74 | 27.10 | 22.76 | 44.04 |
| BMI | Mild | 65 | 29.41 | 4.54 | 29.27 | 20.55 | 41.14 |
| BMI | Moderate | 30 | 29.50 | 4.09 | 29.77 | 22.09 | 41.51 |
| BMI | Severe | 14 | 29.66 | 4.19 | 30.55 | 22.41 | 35.67 |
| ASAT-z | No Apnea | 37 | 0.01 | 1.07 | -0.01 | -1.89 | 2.25 |
| ASAT-z | Mild | 65 | -0.15 | 1.20 | -0.21 | -3.16 | 2.82 |
| ASAT-z | Moderate | 30 | 0.05 | 0.87 | 0.10 | -1.53 | 2.51 |
| ASAT-z | Severe | 14 | -0.10 | 0.56 | -0.19 | -0.93 | 1.11 |
| VAT-z | No Apnea | 37 | 0.59 | 0.94 | 0.53 | -1.34 | 2.64 |
| VAT-z | Mild | 65 | 0.80 | 1.33 | 0.59 | -1.74 | 4.12 |
| VAT-z | Moderate | 30 | 0.79 | 1.25 | 0.42 | -1.68 | 3.68 |
| VAT-z | Severe | 14 | 0.73 | 1.50 | 0.04 | -0.62 | 4.49 |
| TAAT | No Apnea | 37 | 13.34 | 4.87 | 12.40 | 5.31 | 25.18 |
| TAAT | Mild | 65 | 14.40 | 5.18 | 14.27 | 3.70 | 28.71 |
| TAAT | Moderate | 30 | 15.34 | 4.74 | 14.98 | 7.05 | 30.79 |
| TAAT | Severe | 14 | 14.70 | 4.06 | 15.58 | 6.81 | 21.20 |

# **Supplementary Table 2:** Fat distribution and its correlation with Time under 90 % oxygen (T90) and Average saturation (Av Sat), unadjusted and adjusted for BMI

| Sex | Distribution | Variable | β unadj | P unadj | β adj | p adj |
| --- | --- | --- | --- | --- | --- | --- |
| Male | VAT | T90 | 1.7 | 0.0021 | 2.0 | 0.0082 |
| Female | VAT | T90 | 1.8 | 0.079 | 1.2 | 0.32 |
| Male | ASAT | T90 | 0.50 | 0.35 | -0.67 | 0.45 |
| Female | ASAT | T90 | 0.68 | 0.20 | -0.80 | 0.52 |
| Male | TAAT | T90 | 0.66 | 0.028 | 0.79 | 0.17 |
| Female | TAAT | T90 | 0.69 | 0.093 | 0.29 | 0.77 |
| Male | VAT | AvSat | -0.20 | 0.0020 | -0.28 | 0.0029 |
| Female | VAT | AvSat | -0.28 | 0.0076 | -0.30 | 0.018 |
| Male | ASAT | AvSat | -0.023 | 0.72 | 0.15 | 0.18 |
| Female | ASAT | AvSat | -0.046 | 0.41 | 0.079 | 0.55 |
| Male | TAAT | AvSat | -0.068 | 0.063 | -0.086 | 0.22 |
| Female | TAAT | AvSat | -0.072 | 0.092 | -0.14 | 0.17 |

**Supplementary Table 3** Medication Used by Study Participants

| Category | Drug/Treatment Name | Total Patients | Males | Females |
| --- | --- | --- | --- | --- |
|  |  | 164 (100%) | 97 (59.1%) | 67 (40.9%) |
| Glucose Lowering Medications |  |  |  |  |
|  | Metformin | 119 (73.0%) | 75 (77.0%) | 44 (66.0%) |
|  | GLP-1 analogue | 9 (5.5%) | 3 (3.1%) | 6 (9.0%) |
|  | Sulfonylurea | 11 (6.7%) | 6 (6.2%) | 5 (7.5%) |
|  | SGLT2-inhibitor | 43 (26.2%) | 26 (26.8%) | 17 (25.4%) |
|  | DPP-4 inhibitor | 2 (1.2%) | 1 (1.0%) | 1 (1.5%) |
|  | Insulin | 29 (17.7%) | 20 (20.6%) | 9 (13.4%) |
| Antihyperlipidemic Medications |  |  |  |  |
|  | Atorvastatin | 29 (17.7%) | 20 (20.6%) | 9 (13.4%) |
|  | Rosuvastatin | 31 (18.9%) | 13 (13.4%) | 18 (26.9%) |
|  | Simvastatin | 54 (32.9%) | 36 (37.1%) | 18 (26.9%) |
|  | Ezetimibe | 8 (4.9%) | 5 (5.2%) | 3 (4.5%) |
| Antihypertensive Medications |  |  |  |  |
|  | ACE inhibitor | 22 (13.4%) | 15 (15.5%) | 7 (10.4%) |
|  | Angiotensin receptor blocker | 67 (40.9%) | 45 (46.4%) | 22 (32.8%) |
|  | Calcium channel blocker | 30 (18.3%) | 21 (21.6%) | 9 (13.4%) |
|  | Thiazide diuretic | 31 (18.9%) | 18 (18.6%) | 13 (19.4%) |
|  | Loop diuretic | 7 (4.3%) | 5 (5.2%) | 2 (3.0%) |
|  | Beta blocker | 33 (20.1%) | 23 (23.7%) | 10 (14.9%) |
|  | No antihypertensive drug | 42 (25.6%) | 23 (23.7%) | 19 (28.4%) |
